# Supplementary material for: DUCT reveals architectural mechanisms contributing to bile duct recovery in a mouse model for Alagille syndrome
Source: eLife. 2021 Feb 26;10:e60916. doi: 10.7554/eLife.60916 (PMC7909953; doi:10.7554/eLife.60916)
Supplement: Figure 1—figure supplement 6—source data 1. [file elife-60916-fig1-figsupp6-data1.pdf]

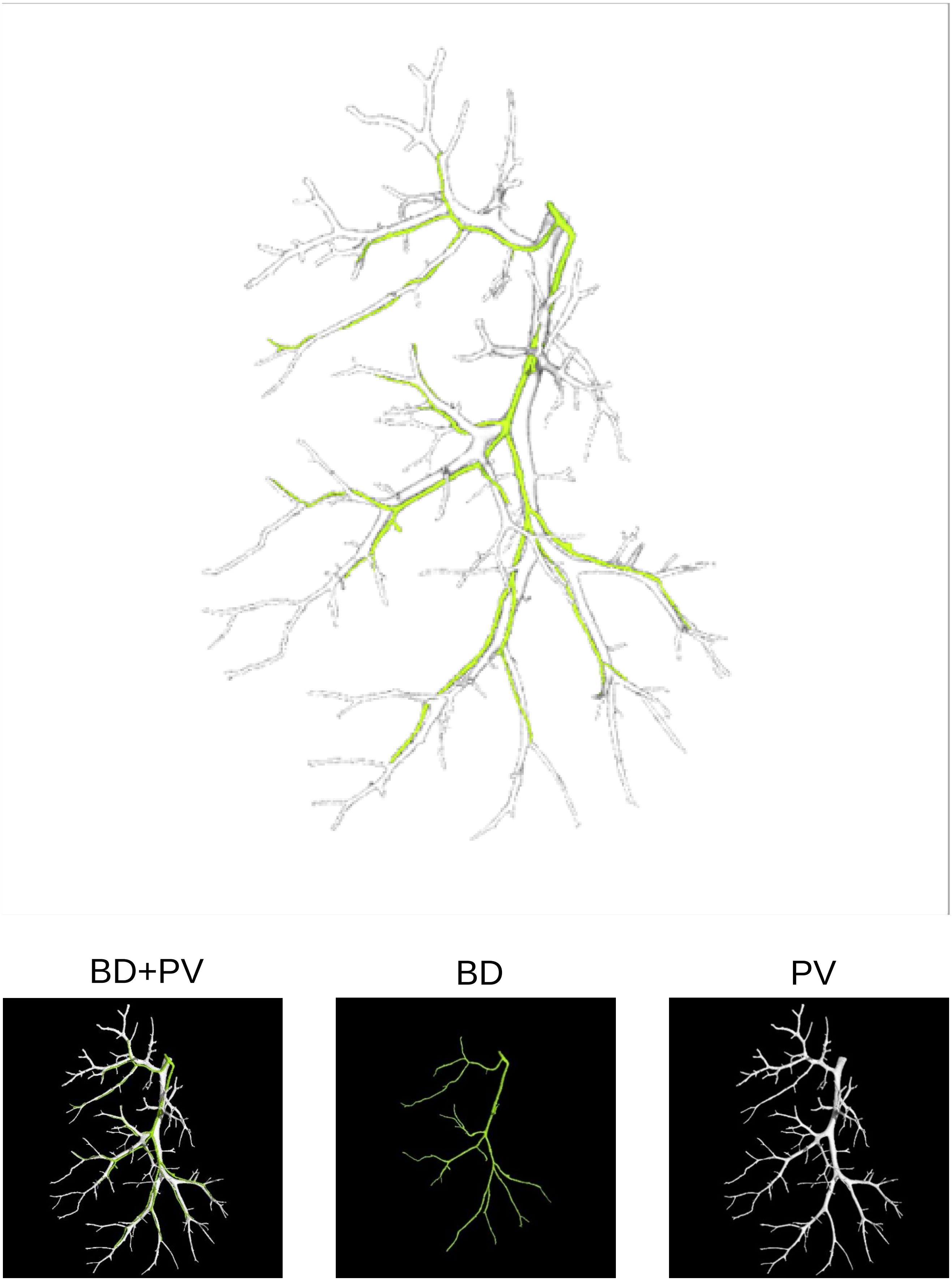

Figure 1 – figure supplement 6. 3D interactive liver cast. Liver cast of adult *Jag1*$^{+/+}$ showing bile duct (green) and portal vein (white) together (top panel) or separately (bottom panels), full rotation, available only online or .pdf.
