# Supplementary material for: DUCT reveals architectural mechanisms contributing to bile duct recovery in a mouse model for Alagille syndrome
Source: eLife. 2021 Feb 26;10:e60916. doi: 10.7554/eLife.60916 (PMC7909953; doi:10.7554/eLife.60916)
Supplement: Transparent reporting form [file elife-60916-transrepform.docx]

***eLife’s* transparent reporting form**

We encourage authors to provide detailed information *within their submission* to facilitate the interpretation and replication of experiments. Authors can upload supporting documentation to indicate the use of appropriate reporting guidelines for health-related research (see [EQUATOR Network](http://www.equator-network.org/%20)), life science research (see the [BioSharing Information Resource](https://biosharing.org/)), or the [ARRIVE guidelines](http://www.plosbiology.org/article/info:doi/10.1371/journal.pbio.1000412) for reporting work involving animal research. Where applicable, authors should refer to any relevant reporting standards documents in this form.

If you have any questions, please consult our Journal Policies and/or contact us: [editorial@elifesciences.org](mailto:editorial@elifesciences.org).

**Sample-size estimation**

- You should state whether an appropriate sample size was computed when the study was being designed
- You should state the statistical method of sample size computation and any required assumptions
- If no explicit power analysis was used, you should describe how you decided what sample (replicate) size (number) to use

Please outline where this information can be found within the submission (e.g., sections or figure legends), or explain why this information doesn’t apply to your submission:

Sample size calculation with the sample size calculator (calculator.net), with a confidence level 95% and confidence interval 80% (± 10%) yields a required sample size 62 per experiment. The estimated sample size is too high for the use of animals in research, and is not practical. The Resource Equation method (Mead 1988) would alternatively have suggested N=6 mice per condition would be suitable.

This study is focused on methods development, thus individual animals were used without sample size estimates for the initial development. For the final experiments with quantifications, we opted for N= 3 per condition to assess which parameters were reproducible. The *Jag1^Ndr/Ndr^* mice display a high degree of heterogeneity in phenotype, and therefore we expect that very large sample sizes would be needed to test for similarity to the wild types for all conditions. We have opted to identify only the most consistently deviant phenotypes, as proof of principle for the method itself.

**Replicates**

- You should report how often each experiment was performed
- You should include a definition of biological versus technical replication
- The data obtained should be provided and sufficient information should be provided to indicate the number of independent biological and/or technical replicates
- If you encountered any outliers, you should describe how these were handled
- Criteria for exclusion/inclusion of data should be clearly stated
- High-throughput sequence data should be uploaded before submission, with a private link for reviewers provided (these are available from both GEO and ArrayExpress)

Please outline where this information can be found within the submission (e.g., sections or figure legends), or explain why this information doesn’t apply to your submission:

The number of biological replicates is described in Material and Method sections, titled Experimental animals and Patient samples.

For quantification of different parameters dot plot graph is used where one dot represents one animal. If a different type of graph is used, in which dot-plots are impractical/obscure the data, the number of animals is described in the figure legend and the raw data is provided in Figure Source Data files.

No outliers were removed from the data based on being “outliers”. Incompletely filled systems were omitted from analyses, determined based on cleared liver lobes, as described in the manuscript.

**Statistical reporting**

- Statistical analysis methods should be described and justified
- Raw data should be presented in figures whenever informative to do so (typically when N per group is less than 10)
- For each experiment, you should identify the statistical tests used, exact values of N, definitions of center, methods of multiple test correction, and dispersion and precision measures (e.g., mean, median, SD, SEM, confidence intervals; and, for the major substantive results, a measure of effect size (e.g., Pearson's r, Cohen's d)
- Report exact p-values wherever possible alongside the summary statistics and 95% confidence intervals. These should be reported for all key questions and not only when the p-value is less than 0.05.

Please outline where this information can be found within the submission (e.g., sections or figure legends), or explain why this information doesn’t apply to your submission:

The statistical analysis is reported in the Material and Method in section titles Statistical analysis. The data is presented within the dot plots. The mean value and SD are displayed in graphs and explained in figure legends. The exact *p* values are reported in the figure legends.

(For large datasets, or papers with a very large number of statistical tests, you may upload a single table file with tests, Ns, etc., with reference to sections in the manuscript.)

**Group allocation**

- Indicate how samples were allocated into experimental groups (in the case of clinical studies, please specify allocation to treatment method); if randomization was used, please also state if restricted randomization was applied
- Indicate if masking was used during group allocation, data collection and/or data analysis

Please outline where this information can be found within the submission (e.g., sections or figure legends), or explain why this information doesn’t apply to your submission:

This manuscript only contains data from wild type and *Jag1^Ndr/Ndr^* mutated mice or healthy controls and patients with Alagille syndrome.

**Additional data files (“source data”)**

- We encourage you to upload relevant additional data files, such as numerical data that are represented as a graph in a figure, or as a summary table
- Where provided, these should be in the most useful format, and they can be uploaded as “Source data” files linked to a main figure or table
- Include model definition files including the full list of parameters used
- Include code used for data analysis (e.g., R, MatLab)
- Avoid stating that data files are “available upon request”

Please indicate the figures or tables for which source data files have been provided:

For Figure 3C - Figure 3 – Source data 1 is available

For Figure 4C - Figure 4 – Source data 1 is available

Figure 1, 3, 4 and 6 where the data was analyzed using custom written matlab pipeline, the code is deposited in Github:

<https://github.com/JakubSalplachta/DUCT>
